# Cases of Intestinal Obstruction, from (Probably) Malarial Causes, with Some Reflections as to a Probable Cause of Intussusception

**Published:** 1875-09

**Authors:** W. D. Hoyt

**Affiliations:** Rome, Georgia


### Cases of Intestinal Obstruction from (probably) Malarial Causes, with some Reflections as to a probable Cause of Intussusception.

By W. D. HOYT, M.D., Rome, Ga.

A series of cases which have recently occurred in my practice, taken in connection with some cases which have presented themselves in the past, have suggested to me some new views as to the cause of certain cases of intestinal obstruction; and the treatment growing out of those views having fortunately been sucessful, I desire to put the cases, and the ideas which they have suggested, on record, with the view of calling the attention of my professional brethren to the subject, and inviting their observation of similar cases.

Cases 1 and 2, Nov. 26th, 1874.—I was called at 3 A.M. to see Mr. W., a large, healthy man, about fifty-five years of age. I found him suffering most intense abdominal pains, the result, he thought, of indigestion, he having drunk freely of buttermilk at supper. He said everything seemed to stop at a certain place in his bowels. The pain was referred to the region of either the stomach or transverse colon. There was no vomiting. The tongue was considerably coated. I gave him a dose of calomel, with opium pills, and applied a hot pepper poultice to the abdomen, and he was relieved. I also directed a dose of oil in the morning, and at my visit on the 27th found that it had acted and he seemed very well.

On the night of the 27th was sent for to see Mrs. B., (case 2d) a lady of fine constitution, but at that time somewhat pulled down, she having been confined on the 31st of October. I was at the time engaged with a very ill child in the country, and did not receive the message until next day, and did not see the patient until 8 P.M. I found she had had a severe attack of what she considered indigestion, and which she attributed to a glass of porter she had taken to increase the secretion of milk. There was no vomiting in this case, only intense pain. The tongue was considerably coated. I found, on inquiry, that she had had a similar but much lighter attack on the night of the 25th. I conceived that both of these cases were of malarial origin, and that Mrs. B.'s case was of the tertian type. I explained to her my views, and told her that the night of the 29th was the time for her to apprehend an attack, which I would endeavor to ward off.

On the 28th, at the same hour as before, 3 A.M., was called again to see Mr. W., and found him suffering from a similar attack. He considered this also indigestion, but could not attribute the attack to any special article of diet, as he had been careful with reference to his food. I pointed out to him the element of periodicity in the case, told him of the other similar case, and told him the attack was not due to indigestion, but probably to a malarial engorgement of the abdominal organs, a recurrence of which we would endeavor to avert with quinine. In the meantime I used opium and a pepper poultice for present relief.

On the 29th Mrs. B. took twelve grains of quinine, so timed as to have her well under the influence of it at night, and as there was a slight manifestation of an attack—enough to confirm the diagnosis—also a little laudanum as directed.

On the 30th, Mr. W. took in the same way fifteen grains of quinine, and escaped the paroxysm entirely. In both cases quinine was used to guard against another paroxysm, and both recovered without further trouble.

Case 3.—On the 1st of December, 1874, I saw an infant child of Capt. C., aged four months, who, as far as I could judge, was suffering from a similar attack. The paroxysms, however, occurred according to my malarial diagram, (concerning which I will hereafter write you,) at noon and midnight. In other words, it was a double quotidian or remittent attack. The brain was much involved through sympathy, and the child was very ill. I used calomel, bromide of potassium and quinine; the latter during both the afternoon and morning remissions, about 6 o'clock, according to the plan I have adopted in malarial attacks of this type, with organic complications. This I propose to elaborate in another article; suffice it now to say, that under this treatment the child made a speedy recovery.

Case 4.—Jan. 27th, 1875, I was called to see Mrs. W., quite a fleshy lady, about 45 years of age. I found that she had been unwell for some time; had been taking Simmons' Liver Medicine freely, and was then suffering great abdominal pain, chiefly referred to the ascending colon. The bowels had not been freely moved. She did not bear opium well. I gave her, therefore, some bromide of potassium, a good dose of blue mass, applied a poultice, and directed her to take, in the morning, in the event of the pill not acting well, a dose of oil and turpentine. 28th.—I was called early to see the patient; she had vomited the oil and turpentine, had had no action from the bowels, and was suffering fearful pain; the abdomen was much distended. Gave opium pills, applied a hot poultice, after rubbing the abdomen with turpentine, and administered copious enemeta of warm water. She obtained no relief, the enemeta failing utterly to move the bowels. Later in the day, gave her two doses of calomel, 15 grains each (the first having been vomited,) desiring to act on the bowels with nature's purgative, bile. She suffered very much all that day and night, the only relief being from the opium. The free enemata administered again and again produced no result, in spite of the free kneading of the abdomen. The next morning—Jan. 29th—I had given her an enema of castor oil, turpentine and tincture of asafœtida, in thin starch, which produced a small, dark, thin action. After this she seemed somewhat easier; but about 2 P.M., I was called again to see her, and found her suffering as much as ever. I repeated the before-mentioned enema, which produced another small action, rather more yellowish in color; after which she was again easier.

About 2 A.M. of the 30th, I was called again to see her, and found her suffering as before; the pain being truly agonizing, and the abdominal tension was as great as ever. Struck with the apparent periodicity in the case, I inquired if the pains did not recur at a definite hour day and night. The intelligent answer was that they recurred regularly at one o'clock, both day and night. I thought this fact, with the evidence of the before-mentioned cases, warranted me in regarding the intestinal obstruction as proceeding from malarial causes. I accordingly wrote a prescription for quinine, four grains to the tablespoonful, in solution with sulphuric acid and water, which I directed the friends to have ready at 6 A.M., gave some opium, applied a hot poultice, and laid down to snatch a short nap, of which I was much in need. Waking between 6 and 7, I gave her, at 7, 9 and 11 A.M., a tablespoonful of the solution. She was free from severe pains all that day, and at 6 and 8 P.M. I gave her another spoonful of the solution. The night was passed pleasantly, and in the course of it and the next day she had several free bilious evacuations, accompanied with much gas, and producing a feeling of great relief. The quinine was continued for another day, when very little additional treatment was necessary; the patient convalescing favorably, though slightly ptyalised. After commencing to take the quinine, the tongue, which was heavily coated, cleared off gradually, and the patient has since been in good health.

The condition producing these symptoms is, I imagine, shown by the next case.

CASE 5. *Obstruction of the bowels, accompanied with great pain; death occurring suddenly; post mortem examination.* In the summer of 1866, soon after coming to Rome, I was called to see Gen. M. L. S., a brave, determined soldier of perhaps forty years of age. He was suffering from constipation of the bowels, accompanied with great pain. A varied treatment was used, combining purgatives with opiates. After using other enemata, a pint of infus. of senna was thrown into the bowel, which produced quite a free action, but brought no feeling of relief.

I have no notes of the case, and at that time my attention had not been directed so closely to the periodicity of diseases. I recollect distinctly, though, that after leaving him quite comfortable late in the evening, I was called up at midnight, and found him suffering great agony. He was, I think, one of the bravest, most self-controlled men I ever knew; but so great was his pain that he could not repress his groans, and the perspiration stood in beads on his forehead. At his request, I called Dr. Battey in to see him with me. He sunk very rapidly, a great change having taken place during my brief absence in going for Dr. Battey, and he soon afterwards died.

As his death had been sudden, and the case peculiar, we deemed a post mortem advisable, expecting to find intussusception. The examination was made by Dr. Battey; Dr. Miller and myself being present. On opening the abdomen, the peritoneum was found intensely congested, both that lining the walls of the abdomen and that covering the viscera. This congestion was much more intense at certain points of the large intestine. There was not a trace of intussusception, but at certain points of the large intestine, and particularly in the descending colon, where the congestion was most intense, there was a contraction of the circular fibres, presenting much the appearance as if a tape was around it. The calibre of the intestine, even in the relaxation of death, was almost obliterated. We imagined that during life the spasmodic contraction of these muscles had completely closed the intestine. The only other pathological condition found was that the gall bladder was filled with gall-stones. Further observations on this case will be made in the closing remarks.

Case 6.—On the 28th of December, 1872, I was called to see Mrs. P., a lady of good constitution, about fifty years of age. Found her suffering from intestinal obstruction, which I endeavored to relieve with opiates and copious enemeta of warm water. After passing into the bowel all the fluid it would hold, she would immediately have an action, passing off at first simply the water thrown in; after a longer or shorter interval another action would follow, bringing away small quantities of thin fæces. Dr. Gorman saw her with me early in her sickness. After using repeated injections for perhaps two days, I procured a stomach tube, which I passed up the rectum, and through which I administered subsequent enemata. On introducing the finger for the purpose of passing this tube, I found the lower portion of rectum much distended, but at a short distance from the sphincter the bowel was so constricted that I could barely introduce the tip of the finger into it. On inserting the end of the tube into this contracted ring and administering an enema, she would have only one action, bringing away small quantities of fæces. It was evident that when administered without the tube the first action was from the portion of bowel below the point of contraction, and that some of the fluid passed through this contracted ring of intestine, producing the second action. Vomiting came on early in this case, and was very aggravated, becoming finally stercoraceous. At one time we applied a blister at the back of the neck, and administered an enema of belladonna and quinine. She seemed to rest decidedly better after this; and, on administering another injection of water through the tube, it was evident that the contracted portion of the bowel was more relaxed. The quinine was used only once a day for two days, when, there being no definite object for using it further, it was laid aside for other remedies. Drs. Battey and G. W. Holmes were in the meantime called in consultation. We considered the question of gastrotomy, but decided against it, as the local symptoms were not well marked. The patient sunk and died on the morning of the 6th of January, 1873. There was much in the physiognomy which strongly suggested the preceding, and of which case 4 afterwards reminded me. No post mortem allowed.

Case 7.—*Malarial spasm of the ascending colon diagnosticated. Recovery under treatment by quinine.*—March 26th, 1875, I was called to see Mrs. E., a lady of about 42 years of age. She was suffering very severe pain, which she supposed to be ovarian.

An examination of the seat of pain revealed the fact that it was located in the right lumbar region, over the ascending colon. Questioning disclosed that on the evening of the 24th she had had some pain; that it had returned on the evening of the 25th with greater severity. She had been easy all day, but the pain had returned with still greater severity shortly before sending for me, about 6 P.M. The bowels were constipated. With the light of the preceding cases I diagnosticated spasm of the ascending colon of the quotidian type. I directed her to take elixir of opium, and to apply a poultice that night, and to take quinine two days. She had no recurrence of the pain, and soon got well.

REMARKS.—I think the malarial origin of five out of seven of these cases is shown by their periodicity, and by the effect of remedies. I may add that the tongue in the worst cases presented in a marked degree the heavy white coat which I have so often seen accompanying intense malarial affections with organic complications. The nature of the attack is, I think, a spasm of the circular muscular fibres of the intestines, produced by derangement in the reflex system of nerves, causing in the worst cases, I think, real intestinal obstruction. This is probably associated with engorgement of the peritoneum, perhaps periodical in character. So far as I have been able to judge the spasm has fallen upon some portion of the large intestine. The intensity of the pain in these cases is a very striking feature. I think the manifestation of pain greater than in any other class of diseases I have witnessed. The idea has also occurred to me that true intussusception may be preceded and produced by this contracted condition of the intestine. Certainly no condition could be so favorable for invagination. Would not contraction *plus* active purgation produce intussusception? May we not have in this condition the explanation of the occurrence of at least some of the cases of intussusception?

Believing that if the ideas here thrown out are correct it is important they should be known, and believing that the patient, in case 4, owed her recovery to the plan of treatment, and that under any other plan of treatment the case would have followed the course of cases 5 and 6, I have prepared this article with the view of calling the attention of my professional brethren to the consideration of these views, and to the observation of similar cases.

